# Osteoporosis-Related Simultaneous Four Joints Fractures and Dislocation after a Seizure: A Case Report

**DOI:** 10.4061/2010/808341

**Published:** 2010-03-03

**Authors:** Abdullah S. AlOmran

**Affiliations:** ^1^College of Medicine, King Faisal University, Dammam, Saudi Arabia; ^2^College of Medicine, King Fahd University Hospital, P.O. Box 40052, Al-Khobar 31952, Saudi Arabia

## Abstract

A case of steroid-induced osteoporosis-related multiple fractures and dislocations are described after a seizure is reported. Patient had two years history of steroid use with no supplement or antiresorptive therapy. There was a delay in the diagnosis which affected an otherwise good outcome in such situations. It is recommended that patients on steroid should be given calcium, vitamin D, and an antiresorptive. Furthermore, a meticulous clinical examination is required in patients who are on steroids and suffer epileptic seizures to rule out skeletal injury.

## 1. Introduction

Prolonged use of corticosteroids cause osteoporosis by both decreasing osteoblastic and increasing osteoclastic activity [1–3]. Skeletal trauma after seizure is well known. However, we are reporting here a case of simultaneous bilateral femoral neck fractures and bilateral humeral neck fractures after one episode of seizure. The patient had two years use of high dose steroid with no antiresorptive therapy. Investigations postinjury documented osteoporosis. This is the first reported simultaneous four axial joints fractures.

## 2. Case Report

**Figure 1:**
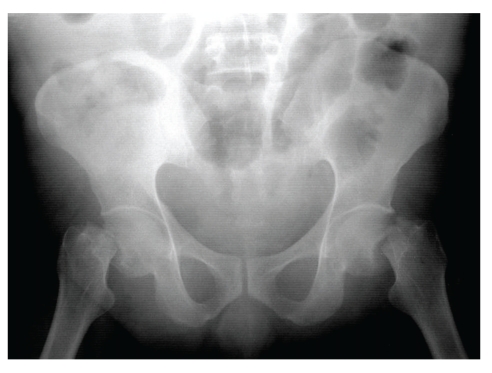
Radiograph of the pelvis showing bilateral fracture of neck of femur.

**Figure 2:**
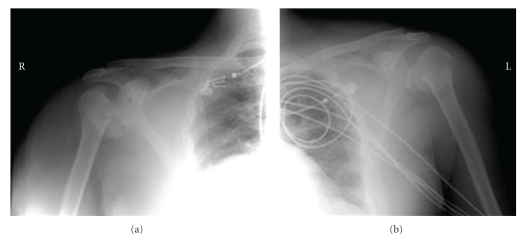
X. ray of both shoulders revealing dislocations.

**Figure 3:**
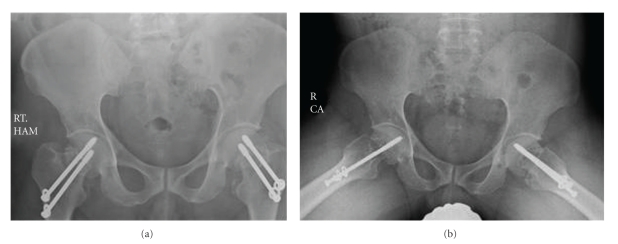
Postfixation of fracture neck of femurs.

A 36 years old Saudi Male was brought to the Emergency Room following a generalized convulsions and loss of consciousness. He was driving a car and felt an acute sharp pain at right shoulder, few seconds later, he developed blurring of vision and he was able to stop the car and lost consciousness for 15–20 minutes. He was observed to have rolling up of the eyes with frothy salivations and trauma to the tongue. The patient complained of severe arthralgia and pain on minimal movement of any joint. On examination he was noted to have petechiae all over his body, no signs of meningeal irritation and there was a lesion over the scapula which was diagnosed as a snake bite. Blood investigations were normal and computerized tomography of the brain was normal. Patient was loaded with phenytoin 25 mg/Kg body weight. In the ward he developed echymotic lesions on the skin and upper extremities, with recurrent vomiting, distended abdomen, paralytic ileus, and deteriorating renal and liver function tests. In the intensive care unit the patient was treated by antivenom and supportive therapy. At the end of the 7th day the general condition improved and patient started to complain of painful hips and shoulders. On further inquiry, patient gave a history of using 15 mg prednisolone daily for aplastic anemia for two years. No supplement or antiresorptive therapy was given to him. Clinical examination showed that both the shoulder joints were dislocated and any attempt to move the hips and right wrist caused tremendous amount of pain. Radiographs showed that bilateral anterior dislocation of shoulder with bilateral fracture surgical neck, Smith's fracture of the right radius and bilateral fracture of the neck of femur (Garden IV) (Figures 1 and 2). Under general anesthesia both shoulder joints were reduced, fracture necks of femur were fixed with two cannulated screws (Figure 3), Smith's fracture was reduced and plaster of paris application was done. Two weeks later, bone mineral density of the spine (Dual Energy X-ray Absorptiometry, DEXA scan) showed severe osteoporosis with T score of −2.9. Last follow-up appointment was four years from the incidence and revealed no pain but rather painless limping. Examination showed right hip and left hip had limited range of movements and pain at extreme degree. The range of movements of the shoulder joints were normal.

## 3. Comments

 Corticosteroids cause low bone mass by cellular apoptosis causing both decreased osteoblastic and increased osteoclastic activity [1]. It is well established that long-term use of glucocorticoid use increases risk of fractures [2, 3]. De Vries et al. [4] reported that a daily dose of higher or equal to 15 milligrams have a substantial higher risk of fractures but Steinbuch et al. [5] believed that higher dose and longer duration is needed to increase the risk of fractures. The guidelines for prevention of Glucocorticoid-induced osteoporosis has been long proposed [6–9]. Devogelaer et al. [10] reported that supplemental calcium and vitamin D should be the first-line therapy in patients receiving ≥7.5 milligrams/daily. Compston [11] suggested that bisphosphonates should be the treatment of choice, with supplementation of calcium and vitamin D. more recent studies [12] suggest teriparatide as a replacement of alandronate. our patient was on 15 mg of prednisolone with no supplementation or antiresorptive therapy.

 Skeletal trauma due to generalized tonic and clonic seizures is not uncommon. Vertebral fractures [13], femoral neck [14], skull [14], shoulder, and humeral head [15] have been reported. Joshy [16] reported a case of bilateral femoral neck fracture due to an epileptic fit. Our patient sustained bilateral femoral neck fractures, bilateral shoulder surgical neck fracture dislocations and a Smith's fracture. It has been reported that fractures are 2–6 times more common in epileptic patients than the general population [17–19]. This has been blamed on low bone mass which is caused by antiepilepsy drugs [20], but our patient sustained all the injuries during the first known attack of the epileptic fit and had steroid-induced low bone mass. 

Bilateral hip fractures are not uncommon and occur due to high energy trauma, and early diagnosis reduces the incidence of permanent disability. Skeletal injuries due to postepileptic fit could be of serious consequences as the diagnosis is often delayed which cause long-term functional disability [21], which in our patient ended in bilateral avascular necrosis of head of femur. Delay in the diagnosis was thought to be due to the fact that patients remain in the intensive care unit, often unconscious and ventilated. Our patient was not ventilated but was complaining of severe arthralgia and difficulty in moving the limbs should have alerted the treating physicians. 

Our case demonstrates that when patients are being treated with long-term steroids, need to be treated with antiresorptives otherwise an episode of epileptic fit could lead to major skeletal trauma.
